# The Book World of Medicine and Science

**Published:** 1895-02-16

**Authors:** 


### THE BOOK WORLD OF MEDICINE AND SCIENCE.

### DISEASES OF THE EYE.

1. A HANDBOOK OF THE DISEASES OF THE EYE AND THEIR TREATMENT. By HENRY R. SWANZY, A.M., M.B, F.R.C.S.I.; edited, under supervision of the Author, by LOUIS WERNER, M.B., B.Ch. (Fifth edition, with illustrations. Small 8vo., pp. 582. London: Lewis. 1895.)
2. LEHRBUCH DER AUGENHEILKUNDE FUR STUDIERENDE UND ÄRZTE. Von. Dr. A. EUGEN FICK. Mit 157 zum teil in Buntdruck ausgeführten Figuren. (8vo., pp. 486. Leipsig: Veit and Co. 1894.)
3. TRAITÉ ÉLÉMENTAIRE D'OPHTHALMOLOGIE. Par H. NIMIER and F. DESPAGNET, avec une planche en couleurs hors texte, et 432 figures dans le texte. (Large 8vo., pp. 944. Paris: Alcan. 1894.)

It is somewhat curious that three text books, one English, one German, and one French, all covering very much the same ground, should be published so nearly at the same time as to fall naturally within the limits of a single notice. The first of the list, the well-known "handbook" of Mr. Swanzy, is, by a few weeks, more recent than either of the others; but the fact that it is in the fifth edition is such a sufficient proof of its merits that a reviewer may almost claim to be relieved from the duty of subjecting it to critical examination. Dr. Fick's treatise may fairly be compared with that of Mr. Swanzy, and contains practically the same information; while that of Drs. Nimier and Despagnet is much more bulky, and on some subjects, especially neurology and refraction, is more full, while on others it is only more diffuse. The authors pride themselves on having drawn their information from French, rather than from foreign sources, saying, "Nous avons suffisamment trouvé en France ce dont nous avions besoin," and it is perhaps for this reason that they make no mention of Von Straub's application of fluorescin to corneal ulcers as a means of discovering their precise extent. This single omission is almost the only important difference which we have discovered between them and their Dublin and Zurich contemporaries.

THE ANATOMY OF THE NASAL CAVITY AND ITS ACCESSORY SINUSES. AN ATLAS FOR PRACTITIONERS AND STUDENTS. By Dr. A. ONODI, Lecturer on Rhino-Laryngology in the University of Budapest. Translated from the second edition by ST. CLAIR THOMSON, M.D.Lond., F.R.C.S.Eng., M.R.C.P.Lond. (London: H. K. Lewis. 1894. Small 4to., pp. 20, plates 16. 6s. net.)

This brochure consists of 20 pages of text, containing a brief description of the walls of the nasal cavities, and of the various sinuses and cells communicating with them. This part contains nothing new, and very little that is not to be found in any good anatomical text-book. Following this are sixteen admirable plates, which have been obtained by engraving carefully-taken photographs of actual sections of the head made in different planes to show every part and relation of the nose and its accessory cavities. The sections have been carefully planned, and the engravings are very accurate and clear. Together they form a very complete and useful atlas of the nose.

CLINICAL MANUAL FOR THE STUDY OF DISEASES OF THE THROAT. By JAMES WALKER DOWNIE, M.B., Maclehose, Glasgow. Illustrated. pp. 265.

In writing this manual it has been the object of the author to meet a want long felt by him in his capacity as a teacher of laryngology. It is divided into two sections. In the first the systematic examination of the parts is discussed; while the second part is devoted to the consideration of the individual diseases. The work is clearly written, the descriptions of the various affections lucid and terse, and the matter well arranged, while the illustrations scattered throughout the text help to render the reader familiar with the aspects assumed by the structures in different pathological conditions. Though in no way competing with the larger and more complete treatises, we feel that this manual will prove a great help to students beginning the clinical study of diseases of the pharynx and larynx, and is one that is sure to be popular.

